# Factors Associated With Psychological Disturbances During the COVID-19 Pandemic: Multicountry Online Study

**DOI:** 10.2196/28736

**Published:** 2021-08-19

**Authors:** Martyna Plomecka, Susanna Gobbi, Rachael Neckels, Piotr Radzinski, Beata Skorko, Samuel Lazzeri, Kristina Almazidou, Alisa Dedic, Asja Bakalovic, Lejla Hrustic, Zainab Ashraf, Sarvin Es Haghi, Luis Rodriguez-Pino, Verena Waller, Hafsa Jabeen, A Beyza Alp, Mehdi Behnam, Dana Shibli, Zofia Baranczuk-Turska, Zeeshan Haq, Salah Qureshi, Adriana M Strutt, Ali Jawaid

**Affiliations:** 1 Neuroscience Center Zurich University of Zurich Zurich Switzerland; 2 Zurich Center for Neuroeconomics University of Zurich Zurich Switzerland; 3 Department of Biomolecular Sciences Boise State University Boise, ID United States; 4 Faculty of Mathematics, Informatics and Mechanics University of Warsaw Warsaw Poland; 5 Medical University of Warsaw Warsaw Poland; 6 Faculty of Science and Engineering University of Groningen Groningen Netherlands; 7 Faculty of Veterinary Medicine Aristotle University of Thessaloniki Thessaloniki Greece; 8 Faculty of Medicine University of Tuzla Tuzla Bosnia and Herzegovina; 9 Faculty of Arts University of Waterloo Waterloo, ON Canada; 10 Faculty of Medicine Shahid Beheshti University of Medical Sciences Tehran Iran; 11 Faculty of Medicine University of Valencia Valencia Spain; 12 Laboratory for Applied Radiobiology Department of Radiation Oncology University Hospital Zurich, University of Zurich Zurich Switzerland; 13 Dow University of Health Sciences Karachi Pakistan; 14 Faculty of Medicine Maltepe University Istanbul Turkey; 15 Faculty of Medicine University of Jordan Amman Jordan; 16 Institute of Mathematics University of Zurich Zurich Switzerland; 17 Texas Behavioral Health Houston, TX United States; 18 Baylor Neurology Baylor College of Medicine Houston, TX United States; 19 Center of Excellence for Neural Plasticity and Brain Disorders: BRAINCITY Nencki Institute of Experimental Biology Warsaw Poland

**Keywords:** COVID-19, pandemic, mental health, depression, posttraumatic stress disorder, general psychological disturbance, global

## Abstract

**Background:**

Accumulating evidence suggests that the COVID-19 pandemic has negatively impacted the mental health of individuals. However, the susceptibility of individuals to be impacted by the pandemic is variable, suggesting potential influences of specific factors related to participants’ demographics, attitudes, and practices.

**Objective:**

We aimed to identify the factors associated with psychological symptoms related to the effects of the first wave of the pandemic in a multicountry cohort of internet users.

**Methods:**

This study anonymously screened 13,332 internet users worldwide for acute psychological symptoms related to the COVID-19 pandemic from March 29 to April 14, 2020, during the first wave of the pandemic amidst strict lockdown conditions. A total of 12,817 responses were considered valid. Moreover, 1077 participants from Europe were screened a second time from May 15 to May 30, 2020, to ascertain the presence of psychological effects after the ease down of restrictions.

**Results:**

Female gender, pre-existing psychiatric conditions, and prior exposure to trauma were identified as notable factors associated with increased psychological symptoms during the first wave of COVID-19 (*P*<.001). The same factors, in addition to being related to someone who died due to COVID-19 and using social media more than usual, were associated with persistence of psychological disturbances in the limited second assessment of European participants after the restrictions had relatively eased (*P*<.001). Optimism, ability to share concerns with family and friends like usual, positive prediction about COVID-19, and daily exercise were related to fewer psychological symptoms in both assessments (*P*<.001).

**Conclusions:**

This study highlights the significant impact of the COVID-19 pandemic at the worldwide level on the mental health of internet users and elucidates prominent associations with their demographics, history of psychiatric disease risk factors, household conditions, certain personality traits, and attitudes toward COVID-19.

## Introduction

The emergence of novel SARS-CoV-2 in December 2019 and the global spread of COVID-19 have become the most severe and publicized human crises in recent history. As of June 29, 2021, the global burden of COVID-19 has exceeded 180 million cases worldwide [1].

The impact of COVID-19 on mental health has recently emerged as a matter of enormous concern [2]. A number of factors related to the pandemic can adversely affect the mental health of individuals, with an even higher risk in those predisposed to psychological conditions [3]. Being in quarantine or isolation for extended periods of time has been associated with depression, anger, anxiety, and suicide as reported in several studies. Similarly, the uncertainty of economic recovery and loss of job security are important factors previously associated with psychological conditions [4-6]. Concerns have also been raised about an increase in the incidents of domestic violence and “screen time” of individuals during the COVID-19 pandemic [7-9], both of which are known risk factors for the development or worsening of psychological conditions [10]. Furthermore, the fear and paranoia of being infected with SARS-CoV-2 and social discrimination could negatively impact mental well-being [11]. The fear of losing a loved one and the grief following loss are other potential disturbances to mental health accompanying serious disease outbreaks [12,13].

Therefore, an assessment of the mental health impact of the COVID-19 pandemic on a global scale is paramount for optimization of mental health services to reduce the long-term morbidity and mortality related to the COVID-19 crisis. Furthermore, this information could aid policymakers in improving the compliance of the general public to lockdown measures [3]. Importantly, COVID-19 and the resulting physical distancing measures have established an unprecedented need to implement and optimize digital mental health services. The experiences and opinions of computer-literate individuals could help in tailoring the services according to their needs, as they are the most likely beneficiaries of digital mental health services [14-16]. The identification of specific individual or community-based vulnerability patterns could also assist in developing strategies to more efficiently deliver mental health services to vulnerable groups. Similarly, by elucidating potential resilience factors that are negatively associated with psychological symptoms, digitally-based strategies could be developed to guide susceptible individuals toward activities that could lessen their distress.

To address this, we assembled a team of health professionals (neuroscientists, psychiatrists, psychologists, data scientists, and medical students) across multiple countries to develop a global online study on the mental health impact of the COVID-19 pandemic. Our first assessment employed a fully anonymous online survey screening individuals in multiple countries for indicators and/or risks of general psychological disturbance, posttraumatic stress disorder (PTSD), and depression. The prevalence of these conditions was then cross-analyzed with participants’ demographics, opinions/outlooks, certain personality traits, current household conditions, previous psychiatric disease history, and factors associated with COVID-19 to identify specific risk and resilience factors. The analysis revealed alarming trends for general psychological disturbances, and risks for PTSD and depression that were specifically associated with participant demographics, personality traits, household conditions, previous psychiatric disease and/or risk factor history, and prediction about COVID-19 resolution. One month later, a limited second assessment was performed targeting European participants when lockdown restrictions had been slightly eased.

## Methods

### Study Design

The study included two assessments separated by 1 month. The first assessment involved a cross-sectional electronic survey–based assessment of individuals above the age of 18 years willing to participate in the study. The anonymous survey was conducted among participants from diverse demographic groups across several continents using standardized self-report scales to screen for general psychological disturbance, risk for PTSD, and symptoms of depression. The survey was available online for a period of 15 consecutive days starting at 6 pm Central European time (CET) on March 29, 2020, and concluding at 6 pm CET on April 14, 2020. The second assessment was performed 1 month after completion of the first assessment for a period of 15 consecutive days starting at 6 pm CET on May 15, 2020, and concluding at 6 pm CET on May 30, 2020. The second assessment was limited to European participants, and the participants were asked to fill the survey only if they had completed the first assessment.

### Questionnaire Development

The questionnaire was developed via close consultation among a neuroscientist, a neuropsychologist, a psychiatrist, a data scientist, and a psychiatry clinic manager. The questionnaire included closed-ended questions that assessed participant characteristics and opinions, and screened for psychological conditions through standardized and validated self-report scales. The questionnaire prototype was prepared in English (Multimedia Appendix 1) and translated into 10 additional languages (Arabic, Bosnian, French, German, Greek, Italian, Persian, Polish, Spanish, and Turkish) by bilingual native speakers and vetted by volunteers native to those countries. The feasibility of each questionnaire was confirmed using pilot studies of 10 participants each. These responses were excluded from the final analysis.

The questionnaires (Multimedia Appendix 1) included a section on participant demographics (age, gender, country, residential setting, educational status, and current employment status), household conditions (working/studying from home, home isolation conditions, pet ownership, level of social contact, social media usage, and time spent exercising), COVID-19–related factors (knowing a co-worker, friend, or family member who tested positive for COVID-19 or was thought to have died due to COVID-19, and prediction about pandemic resolution), certain personality traits (level of optimism and level of extroversion), history of psychiatric disease and/or trauma, previous exposure to human crisis, and levels of satisfaction with the actions of the state and employer during the current crisis. All questionnaires were rated on binary (yes/no) responses or Likert-type scales.

The other sections contained assessments based on the World Health Organization (WHO) Self-Reporting Questionnaire-20 (SRQ), Impact of Event Scale (IES), and Beck Depression Inventory II (BDI) [17-19]. These scales were chosen based on their common usage and efficacy in previously employed work studying the psychological impact of human crises including the SARS epidemic [20-29]. The IES was purposefully adjusted to assess the impact of an ongoing event rather than a past event. For this purpose, the past tense was converted to the present tense in each question without changing the subject matter. This adjustment was performed in consultation with an independent neuropsychologist not involved in the study. For all scales, participants were prompted to think of and report their physical and psychological states during the preceding week. The second assessment was only limited to the SRQ.

### Ethical Considerations

Informed consent was obtained from each participant to allow for anonymous recording, analysis, and publication of their answers. The data were collected in a completely anonymous fashion without recording any personal identifiers, ensuring that the confidentiality of the participants was maintained in all phases of the study. The study procedures were reviewed and approved by the University of Zurich Research Office for Scientific Integrity and Cantonal Ethics Commission for the canton of Zurich (Switzerland; Multimedia Appendix 2), BRAINCITY Centre of Excellence for Neural Plasticity and Brain Disorders, Nencki Institute of Experimental Biology, Warsaw (Poland; Multimedia Appendix 3), and Faculty of Medicine, University of Tuzla, Tuzla (Bosnia and Herzegovina; Multimedia Appendix 4).

### Data Collection

#### First Assessment

Using a nonrandomized referral sampling (snowball sampling) method, participants were contacted by a team of 70 researchers of diverse nationalities (study authors and volunteers who have been acknowledged in the Acknowledgment section) via electronic communication channels that included posts on social media platforms, direct digital messaging, and personal and professional email lists. For this assessment, the data collection procedures were repeated at least thrice during the data collection period (March 29 to April 14, 2020).

The data collection strategy resulted in a total of 13,332 responses during the first assessment. Surveys in which participants were younger than 18 years (n=34), responses were missing for any dependent variables (n=112), individuals had participated a second time (n=325), and geographic location was missing (n=20), as well as those that originated from the WHO African region (n=24) were excluded from the final analysis. When the responses were missing for individual items, the missing data were considered null and excluded from the analysis for that particular variable. The number of participants for 12 featured countries and the regions encompassing the other countries is represented in Multimedia Appendix 5.

#### Second Assessment

For the second assessment, data collection was limited to European participants only. The data collection team from Europe called upon potential participants using the same electronic communication channels that were used for data collection during the first assessment. The participants were prompted to fill the survey only if they had previously completed the first assessment. Data collection procedures were repeated three times during the data collection period, resulting in a total of 1077 responses during the second assessment. Against the 6207 responses collected from Europe during the first assessment, this established a response rate of 17.35%.

### Statistical Analysis

All statistical analyses were performed using R version 3.6.3 and *Rstudio* [30]. All figures were produced using the packages *ggplot2* [31] and *CGPfunctions* [32].

#### Nonadjusted Analysis for SRQ, IES, and BDI scores

Mean scores with standard deviations were calculated for the SRQ, IES, and BDI from all valid responses (n=12,817) and compared across all of the below categorical factors via Kruskal-Wallis tests with the chi-square function. The categorical factors included gender, residential status, education level, employment status, being a medical professional, working remotely from home, satisfaction with the response of the employer to the pandemic, satisfaction with the response of the state (country government) to the pandemic, home isolation status, level of interaction with family and friends, social media usage, ability to share concerns with a mental health professional, ability to share concerns with family and friends, prior exposure to a human crisis situation, previous exposure to trauma, level of extroversion, optimism about COVID-19 resolution, and one’s self-determined role in the pandemic.

#### Multiple Regression Models for the SRQ, IES, and BDI

Multiple linear and logistic regression models were built for the SRQ, IES, and BDI, using mean scores and cutoffs for respective categorical classification.

For linear regression, generalized linear models with the *glm* function were devised using the *lme4* package [33]. The three univariate linear regression models, one each for the SRQ, IES, and BDI, were fitted and corrected for multiple comparisons followed by *glm* function analyses. Following Bonferroni correction for multiple comparisons, the *P*-value threshold was set to *P*=.017. For each linear regression model, “age” was entered as a continuous independent predictor, whereas all aforementioned predictors were entered as categorical fixed effects. Poisson family and log link function were used to model BDI and SRQ factors. In order to choose the best model (based on Akaike information criterion [AIC] or Bayesian information criterion [BIC]) from the set of predictors, stepwise model selection was performed from the *MASS* package [34].

Logistic regression was performed to generate odds ratios (ORs) for the SRQ, IES, and BDI using the following categorization scheme: SRQ: 0=normal (0-7 points), 1=concern for general psychological disturbance (8-20 points); IES: 0=normal (0-23 points), 1=PTSD is a clinical concern (24-32 points), 2=threshold for a probable PTSD diagnosis (33-36 points), 3=severe condition (high enough to induce immunosuppression) (≥37 points), and for generating ORs, the variables were regrouped as 0=no concern versus any type of concern (levels 1/2/3); BDI: 0=these ups and downs are considered normal (1-10 points), 1=mild mood disturbance (11-16 points), 2=borderline clinical depression (17-20 points), 3=moderate depression (21-30 points), 4=severe depression (31-40 points), 5=extreme depression (>40 points), and for generating ORs, the variables were regrouped as 0=no concern versus any type of concern (levels 1/2/3/4/5). Cutoffs for the SRQ, IES, and BDI were defined using least stringent thresholds for each of these measures from previous literature to ensure high sensitivity of the screening [17-20,35]. Furthermore, separate OR analysis was performed with the reference level set to 0=absence of symptom compared to presence of symptom (varying severity levels of the symptom regrouped into one category). Correlations among the SRQ, IES, and BDI were assessed through the Pearson correlation test and illustrated as x-y plots.

For the second assessment, a generalized linear model with the *glm* function was fitted using the *lme4* package [33]. All predictors were entered as categorical fixed effects. Poisson family and log link function were used to model the SRQ factor. An interaction effect was introduced to inspect whether the second assessment and working from home, satisfaction with the employer, having a pre-existing psychiatric condition, closely knowing someone who died of COVID-19, and residence (urban or rural) had a significant effect on SRQ score progression during the first and second assessments.

## Results

### First Assessment

**Figure 1:**
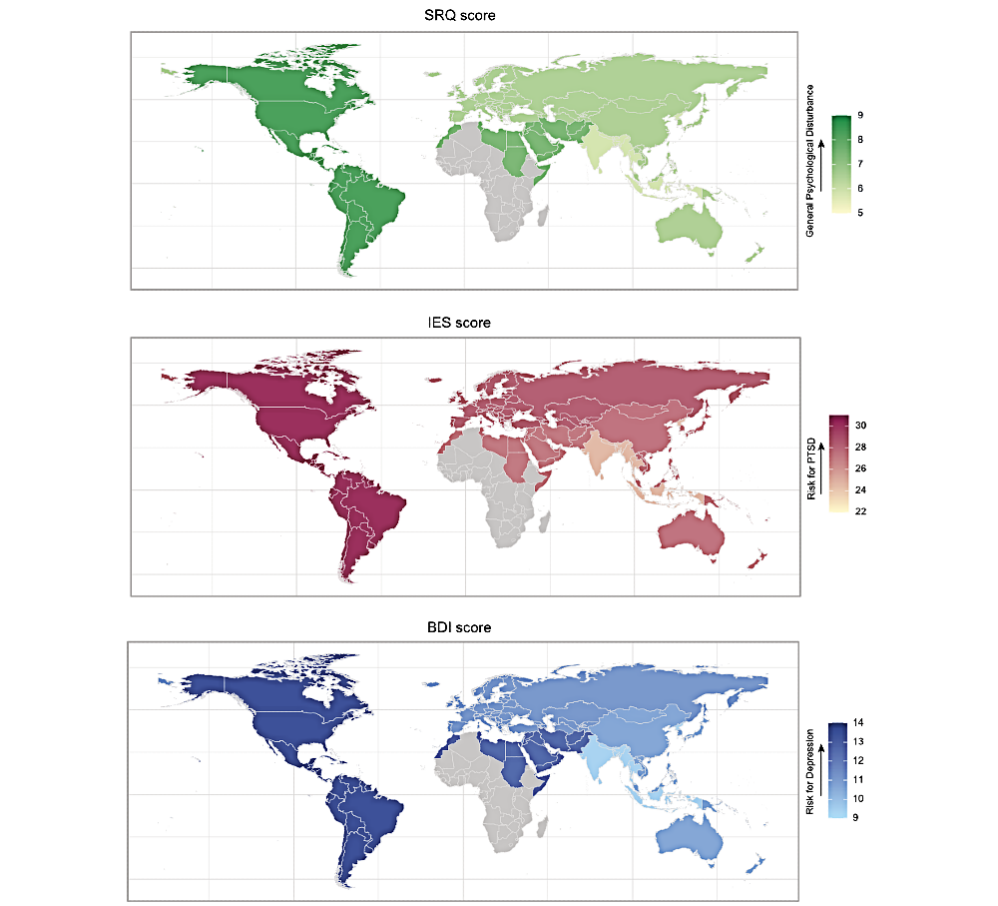
Geodemographic representation of global mental health burden. The three maps present mean scores from the World Health Organization (WHO) Self-Reporting Questionnaire-20 (SRQ), Impact of Event Scale (IES), and Beck Depression Inventory II (BDI). The means were calculated separately for each of the countries and for each WHO region. The total number of responders is 12,817. First panel: mean scores for the SRQ, indicating general psychological disturbance; Second panel: mean scores for the IES, indicating risk for posttraumatic stress disorder (PTSD); Third panel: mean scores for the BDI, indicating risk for depression. All mean scores were calculated separately for the featured countries and WHO regions.

A total of 12,817 valid responses were divided across the United States (n=1864), Iran (n=1198), Pakistan (n=1173), Poland (n=1110), Italy (n=1096), Spain (n=972), Bosnia and Herzegovina (n=885), Turkey (n=539), Canada (n=538), Germany (n=534), Switzerland (n=489), and France (n=337). The remaining countries were grouped according to WHO regions, that is, the European region (EURO; n=784), East Mediterranean region (EMRO; n=459), Western Pacific region (WPRO; n=326), South East Asian region (SEARO; n=259), and region of the Americas (PAHO; n=254). Overall, a prominent psychological impact of COVID-19 was evident worldwide with the highest SRQ scores (indicating general psychological disturbance) in Bosnia and Herzegovina, Canada, Pakistan, and the United States, and highest IES (indicating risk of PTSD) and BDI (indicating risk of depression) scores in Canada, Pakistan, and the United States (Figure 1).

There was an evident disproportion in valid responses overall, with higher numbers from those participants who reported being female (n=9314, 72.4%), residing in urban areas (n=10,666, 82.9%), having an advanced educational qualification (ie, bachelor’s degree or higher) (n=9653, 75.0%), working/studying remotely from home (n=8289, 64.4%), and being under home isolation with a partner/family (n=10,691, 83.1%). Moreover, of notable prevalence were factors such as expressing satisfaction with the COVID-19–related employer response (n=4364, 33.9%), being somewhat satisfied with the COVID-19–related state response (n=4772, 37.1%), and spending less than 15 minutes on daily physical exercise (n=6306, 49.0%). A majority of participants also reported increased social media usage (n=8385, 65.1%), less than usual or minimal interaction with family and friends (n=7723, 60.0%), and feeling a sense of control in protecting themselves and others during the COVID-19 pandemic (n=10,408, 80.9%). Details of participant demographics, household conditions, history of psychiatric conditions, previous exposure to trauma/crisis, personality traits, and COVID-19–related factors and opinions are presented in Multimedia Appendix 6.

### Unadjusted Analysis of Risk and Resilience Factors for General Psychological Disturbance (SRQ), PTSD Risk (IES), and Depression (BDI)

Unadjusted analyses of SRQ, IES, and BDI scores between different participant demographics/characteristics showed a significantly greater prevalence (*P*<.017) of psychological symptoms in participants who were female, unemployed, working remotely from home, dissatisfied with the response of their employer/state to COVID-19, home isolated alone or with a pet, interacting with friends/family less than usual, and using social media more than usual, as well as those with a less than usual ability to share concerns with friends/family. Significantly higher scores (*P*<.017) on the SRQ, IES, and BDI were also seen in participants who self-reported being a pessimist or introvert, not feeling in control during COVID-19, and having an overall negative prediction about COVID-19 resolution. The means and standard deviations for all comparisons are presented in Table 1.

**Table 1:** Comparison of psychological symptoms between different participant demographics/characteristics.

Factor			Score^a^, mean (SD)			
			SRQ^b^		IES^c^	BDI^d^
**Gender**						
	Male	5.29 (4.64)^e^		23.57 (14.06)^e^		9.17 (9.07)^e^
	Female	7.62 (5.05)^e^		30.22 (14.16)^e^		12.88 (10.05)^e^
	Nonbinary	9.98 (5.87)^e^		34.18 (16.81)^e^		18.58 (11.78)^e^
	Not disclosed	7.09 (5.32)^e^		27.78 (15.80)^e^		13.11 (10.61)^e^
**Residence**						
	Rural	6.88 (5.08)		28.07 (14.58)		11.74 (9.60)
	Urban	7.08 (5.06)		28.63 (14.43)		12.04 (10.04)
**Education**						
	Compulsory	7.05 (5.09)^e^		27.64 (14.58)^e^		12.56 (10.51)^e^
	Advanced	7.05 (5.07)^e^		28.87 (14.42)^e^		11.84 (9.81)^e^
**Work status**						
	Private employed	6.35 (4.84)^e^		26.54 (14.05)^e^		10.30 (9.02)^e^
	Public employed	6.63 (5.17)^e^		28.22 (14.71)^e^		11.02 (9.56)^e^
	Freelancer	6.30 (4.81)^e^		27.19 (14.42)^e^		10.67 (9.32)^e^
	Unemployed	8.14 (5.26)^e^		29.90 (15.07)^e^		13.96 (11.12)^e^
**Medical or health care professional**						
	No	7.09 (5.09)^e^		28.61 (14.44)		12.12 (10.04)^e^
	Yes	6.50 (4.87)^e^		28.01 (14.89)		10.76 (9.19)^e^
**Remotely working from home**						
	No	6.63 (5.01)^e^		27.60 (14.88)^e^		11.70 (10.10)^e^
	Yes	7.25 (5.08)^e^		29.04 (14.22)^e^		12.15 (9.91)^e^
**Opinion about employer response to COVID-19**						
	Not satisfied	8.70 (5.22)^e^		32.39 (15.24)^e^		15.18 (11.31)^e^
	Somewhat satisfied	7.64 (5.01)^e^		29.80 (14.18)^e^		12.71 (9.76)^e^
	Satisfied	5.92 (4.83)^e^		26.42 (14.15)^e^		9.83 (8.99)^e^
**Opinion about state response to COVID-19**						
	Not satisfied	7.78 (5.14)^e^		30.83 (14.76)^e^		13.74 (10.66)^e^
	Somewhat satisfied	7.08 (4.96)^e^		28.55 (13.88)^e^		11.89 (9.42)^e^
	Satisfied	6.25 (5.00)^e^		26.31 (14.48)^e^		10.37 (9.61)^e^
**Home isolation**						
	Not isolated	5.29 (4.58)^e^		25.20 (14.68)^e^		9.44 (9.01)^e^
	Individual home isolation	7.68 (5.37)^e^		30.04 (15.15)^e^		13.25 (10.58)^e^
	Home isolation with family or partner	7.14 (5.05)^e^		28.70 (14.34)^e^		12.10 (9.97)^e^
**Presence of a pet at home**						
	No pet at home	6.81 (5.00)^e^		27.92 (14.37)^e^		11.55 (9.85)^e^
	Pet at home	7.48 (5.16)^e^		29.74 (14.57)^e^		12.85 (10.16)^e^
**Interaction with family or friends**						
	Less than usual	7.57 (5.02)^e^		29.77 (14.18)^e^		12.62 (9.87)^e^
	Minimal interaction	7.34 (5.26)^e^		28.69 (14.69)^e^		12.74 (10.64)^e^
	Like usual	6.41 (4.89)^e^		27.45 (14.38)^e^		10.89 (9.42)^e^
**Use of social media**						
	Less than usual	7.61 (5.37)^e^		29.89 (16.06)^e^		13.47 (11.42)^e^
	Like usual	5.56 (4.70)^e^		25.28 (14.20)^e^		10.17 (9.33)^e^
	More than usual	7.64 (5.07)^e^		29.89 (14.22)^e^		12.69 (10.03)^e^
**Time dedicated to physical exercise**						
	Less than 15 minutes	7.70 (5.17)^e^		29.33 (14.82)^e^		13.22 (10.61)^e^
	More than 15 minutes	6.65 (4.90)^e^		28.26 (13.91)^e^		11.06 (9.04)^e^
	More than 1 hour	5.72 (4.75)^e^		26.56 (14.30)^e^		10.06 (9.27)^e^
**Close person positive for COVID-19**						
	No	6.97 (5.09)^e^		28.25 (14.55)^e^		12.00 (10.07)
	Yes	7.26 (5.02)^e^		29.43 (14.16)^e^		12.01 (9.71)
**Close person died due to COVID-19**						
	No	7.04 (5.08)		28.53 (14.52)		12.00 (9.99)
	Yes	7.07 (4.95)		28.71 (13.67)		11.76 (9.81)
**Psychiatric condition**						
	No psychiatric condition	6.21 (4.70)^e^		26.80 (13.88)^e^		10.34 (8.83)^e^
	No change in pre-existing psychiatric condition	6.16 (4.31)^e^		25.74 (13.14)^e^		10.63 (8.53)^e^
	Worsening of pre-existing psychiatric condition	12.5 (4.12)^e^		40.57 (12.84)^e^		22.53 (10.75)^e^
**Ability to share concerns with a health professional**						
	No	8.44 (5.16)^e^		31.79 (14.46)^e^		14.50 (10.74)^e^
	Yes	7.52 (5.11)^e^		30.09 (14.87)^e^		12.88 (10.35)^e^
**Ability to share concerns with family or friends**						
	No	9.32 (5.69)^e^		31.59 (16.29)^e^		17.87 (13.25)^e^
	Less than usual	9.78 (4.99)^e^		34.68 (14.23)^e^		17.06 (10.60)^e^
	Like usual	5.95 (4.59)^e^		26.37 (13.67)^e^		9.78 (8.35)^e^
**Previous exposure to a crisis**						
	No	7.05 (5.02)		28.52 (14.21)		11.99 (9.92)
	Yes	7.03 (5.20)		28.79 (15.12)		12.11 (10.15)
**Previous exposure to traumatic experiences**						
	No	6.21 (4.75)^e^		26.40 (14.05)^e^		10.46 (9.07)^e^
	Yes	8.03 (5.30)^e^		31.48 (14.80)^e^		13.99 (10.87)^e^
	Yes (before the age of 17 years)	7.81 (5.10)^e^		29.57 (13.87)^e^		12.92 (10.04)^e^
**Personality type**						
	Extrovert	6.36 (4.89)^e^		27.49 (14.36)^e^		10.42 (9.09)^e^
	Introvert	7.65 (5.16)^e^		29.05 (14.42)^e^		13.16 (10.45)^e^
**Personality**						
	Pessimist	9.99 (4.98)^e^		34.89 (14.46)^e^		18.41 (11.23)^e^
	Optimist	5.57 (4.62)^e^		25.81 (13.81)^e^		8.86 (7.92)^e^
	Realist	7.33 (4.98)^e^		28.86 (14.24)^e^		12.61 (9.95)^e^
**Prediction about COVID-19 outcome/resolution**						
	It might be the end of the human race	10.00 (5.42)^e^		38.41 (16.48)^e^		21.88 (13.85)^e^
	It will resolve after many months or years	7.81 (5.20)^e^		30.62 (14.92)^e^		13.64 (10.68)^e^
	It will resolve in the summer but not within a month	6.76 (4.93)^e^		27.94 (13.93)^e^		11.23 (9.41)^e^
	It will resolve within a month	6.36 (5.21)^e^		26.63 (14.80)^e^		10.62 (9.70)^e^
**Self-opinion in the COVID-19 pandemic**						
	It is not in my control at all	10.11 (5.39)^e^		34.77 (16.50)^e^		18.65 (13.70)^e^
	It is not in my control, but I can take precautions to protect myself	7.83 (5.30)^e^		30.39 (15.23)^e^		13.45 (10.69)^e^
	It is not in my control, but I can take precautions to protect myself and others	6.77 (4.96)^e^		28.03 (14.10)^e^		11.48 (9.51)^e^

^a^The scores are divided according to different participant demographics/characteristics and compared through unadjusted Kruskal-Wallis tests.

^b^SRQ: Self-Reporting Questionnaire-20.

^c^IES: Impact of Event Scale.

^d^BDI: Beck Depression Inventory II.

^e^Significant differences (*P* value threshold set to *P*<.017 after multiple comparison correction) in mean scores are indicated. Each association indicates a difference in categories reported in the column vertically.

### Adjusted Analysis of Factors Associated With General Psychological Disturbance (SRQ), PTSD Risk (IES), and Depression (BDI)

**Figure 2:**
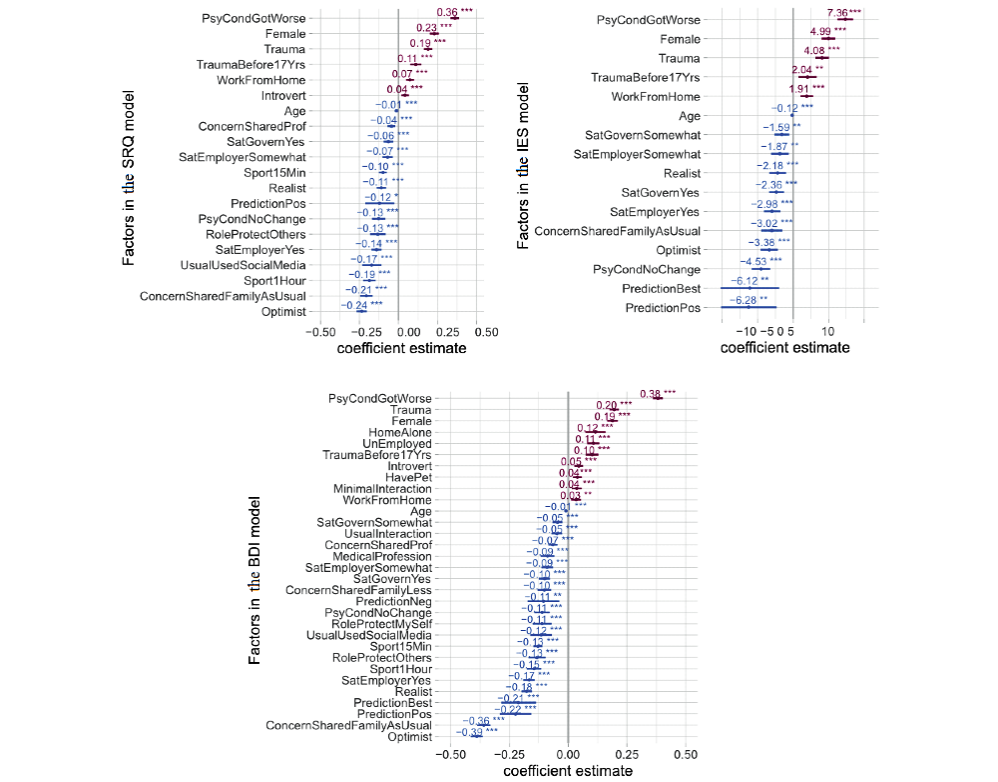
Risk and resilience factors for general psychological disturbance (Self-Reporting Questionnaire-20 [SRQ]), risk for posttraumatic stress disorder (PTSD) (Impact of Event Scale [IES]), and depression (Beck Depression Inventory II [BDI]). These foster plots show the mean estimates and the 95% CIs for adjusted coefficients significantly affecting SRQ, IES, and BDI scores generated through multiple regression models. Only factors that survived Bonferroni correction for multiple comparisons (*P*<.017) are listed. Factors associated with an increase in scores are shown in red, while those associated with a decrease in scores are in blue.

Adjusted analysis using different general linear models for each of the questionnaires is reported in Figure 2. Across all three questionnaires, we found the following factors increasing general psychological disturbance, PTSD, and depression: a psychiatric condition that worsened during the COVID-19 pandemic (SRQ mean coefficient: 0.36, 95% CI 0.33-0.39; IES mean coefficient: 7.36, 95% CI 6.26-8.46; BDI mean coefficient: 0.38, 95% CI 0.36-0.40), previous exposure to trauma (SRQ mean coefficient: 0.19, 95% CI 0.16-0.22; IES mean coefficient: 4.08, 95% CI 3.14-5.03; BDI mean coefficient: 0.20, 95% CI 0.17-0.22), and working remotely from home (SRQ mean coefficient: 0.07, 95% CI 0.05-0.10; IES mean coefficient: 1.91, 95% CI 1.01-2.82; BDI mean coefficient: 0.03, 95% CI 0.01-0.05).

Moreover, significant gender differences were observed, with higher risk in women versus men for general psychological disturbances (SRQ mean coefficient: 0.23, 95% CI 0.20-0.26), PTSD (IES mean coefficient: 4.99, 95% CI 4.03-5.95), and depression (BDI mean coefficient: 0.19, 95% CI 0.17-0.21).

**Figure 3:**
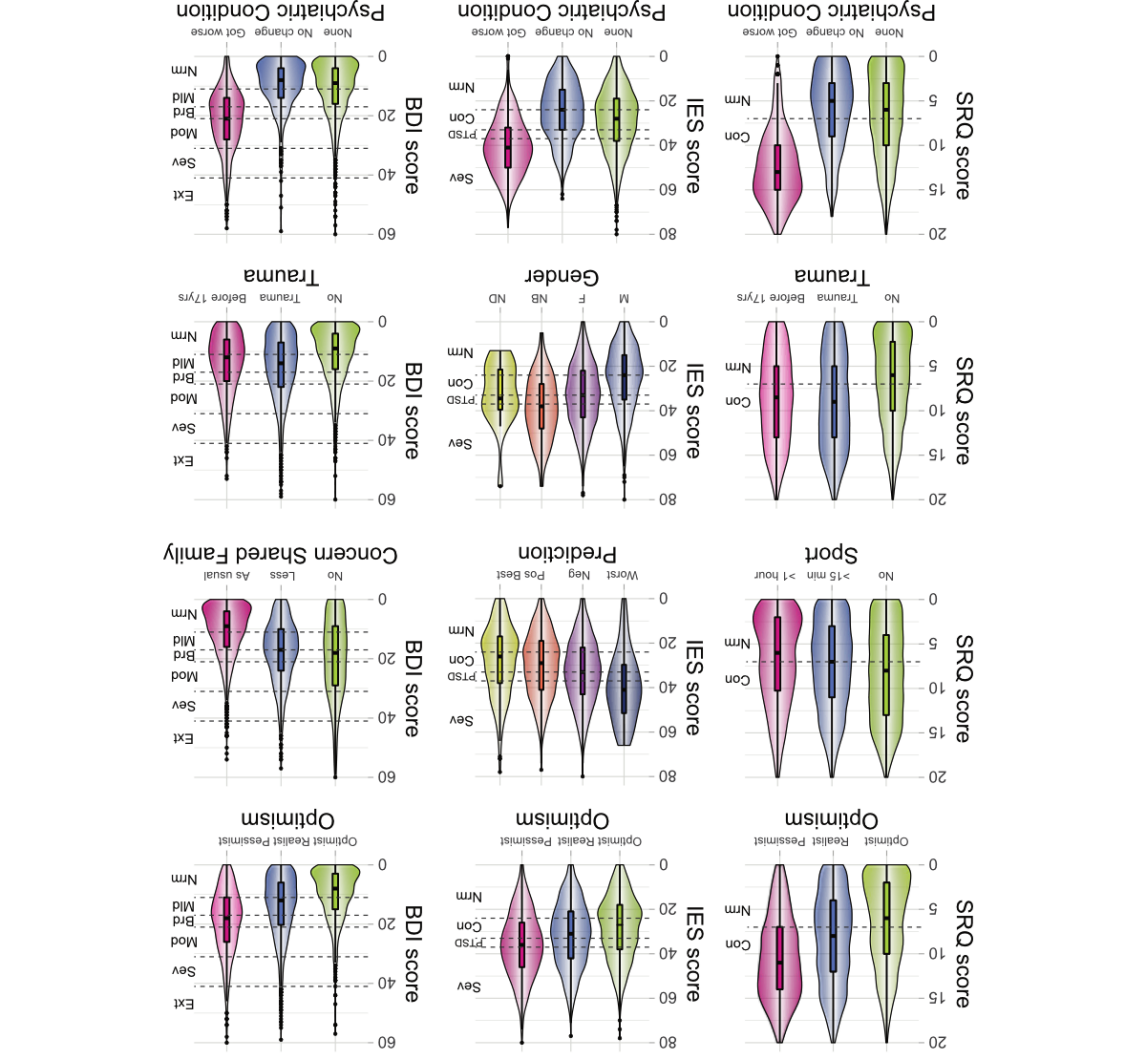
Violin plots indicating the effects of selected factors on general psychological disturbance (Self-Reporting Questionnaire-20 [SRQ]), risk for posttraumatic stress disorder (PTSD) (Impact of Event Scale [IES]), and depression (Beck Depression Inventory II [BDI]). These plots provide a relation between the participant scores on the SRQ, IES, and BDI and participant characteristics (previous history of a psychiatric condition, past exposure to trauma, prediction about COVID-19 resolution, level of optimism, gender, and daily physical activity/sports) adjusted for confounding variables through multiple regression models. Boxplots display the distribution of the selected factors with the visualization of five summary statistics (minimum, maximum, median, first quartile, and third quartile), and all outliers individually. Violin plots added behind the boxplots visualize the probability density of selected factors. Parallel to the x-axis, dashed lines present cutoffs for the scales used. For the BDI, Ext is “extreme,” 40+ points, extreme depression; Sev is “severe,” 31-40 points, severe depression; Mod is “moderate,” 21-30 points, moderate depression; Brd is “borderline,” 17-20 points, borderline clinical depression; Mld is “mild,” 11-16 points, mild mood disturbance; and Nrm is “normal,” 1-10 points, considered normal. For the SRQ, Con is “concern,” 8-20 points, clinical concern for general psychological disturbance and Nrm is “normal,” 0-7 points. For the IES, Sev is “severe,” 37+ points, symptoms high enough to suppress the immune system; PTSD is “posttraumatic stress disorder,” 34-36 points; Con is “clinical concern for possible PTSD,” 24-33 points; and Nrm is “Normal,” 0-23 points.

Having an optimistic attitude, having a positive prediction about COVID-19, and being able to share concerns with family/friends decreased SRQ, IES, and BDI scores, indicating the protective effect of these factors against general psychological disturbance, PTSD, and depression (as shown in Figure 2 and Figure 3). Furthermore, daily physical activity/sports decreased both SRQ (mean coefficient: −0.19, 95% CI −0.23 to −0.15) and BDI (mean coefficient: −0.15, 95% CI −0.18 to −0.12) scores, with greater reductions resulting from the duration of physical activity/sports (exercise for ≥1 hour was more effective in decreasing SRQ and BDI scores compared to exercise for >15 minutes but <1 hour). In addition, health care professionals reported significantly lower BDI scores compared to nonhealth care professionals, suggesting this status to have a protective effect against depression (mean coefficient: −0.09, 95% CI −0.12 to −0.06).

The logistic regression analyses performed after classifying SRQ, IES, and BDI scores into categorical cutoffs confirmed the primary results from the linear regression models (Multimedia Appendix 7). An individual with pre-existing psychiatric conditions that worsened during COVID-19 showed seven times higher odds of being depressed (OR 7.10, 95% CI 6.03-8.35), 1.6 times higher odds of having PTSD (OR 1.60, 95% CI 1.38-1.84), and two times higher odds of having general psychological disturbance (OR 2.64, 95% CI 1.99-3.48). As expected, individuals with previous trauma exposure exhibited greater ORs than their counterparts for these conditions according to the BDI (OR 1.61, 95% CI 1.46-1.76) and SRQ (OR 2.62, 95% CI 2.08-3.30). Still, an optimistic attitude and the opportunity to share concerns with family/friends like usual served as protective factors for general psychological disturbance according to the SRQ (OR 0.51, 95% CI 0.43-0.62 and OR 0.19, 95% CI 0.15-0.23, respectively) and depression according to the BDI (OR 0.23, 95% CI 0.20-0.26 and OR 0.39, 95% CI 0.33-0.45, respectively).

For visual aid, the association of participant-related factors with categorical classifications for general psychological disturbance (SRQ), PTSD (IES), and depression (BDI) are indicated through box plots in Multimedia Appendix 8. Owning a pet, having a pre-existing psychiatric condition, having previous exposure to trauma, considering oneself an introvert, and working remotely from home were associated with decreased percentages of responses in the unaffected (“normal”) category based on the SRQ, IES, and BDI, suggesting these as risk factors. In contrast, a majority of responses from health care professionals landed in the unaffected (“normal”) category for the BDI.

### Correlation Among Scales

The continuous scores of all responses on the SRQ, BDI, and IES were also analyzed by Pearson correlations using all possible combinations on x-y plotting (SRQ vs IES, IES vs BDI, and BDI vs SRQ). All combinations yielded significant correlations, with the strongest correlation (R=0.79) between the BDI and SRQ (Multimedia Appendix 9).

### Second Assessment

The demographic distribution of European participants included in the second assessment was similar to that in the first assessment, with higher number of responses from those participants who were female (n=803, 74.6%), working/studying remotely from home (n=613, 56.9%), and currently under home isolation with a partner/family (n=703, 65.3%). A majority of participants also reported increased social media usage (n=667, 61.9%), less than usual or minimal interaction with family and friends (n=703, 65.3%), and feeling a sense of control in protecting themselves and others during the COVID-19 pandemic (n=666, 61.9%).

Unadjusted analyses of SRQ scores between different participant demographics/characteristics showed a significantly higher prevalence of psychological symptoms (*P*<.05) in participants who were female, medical or health care professionals, dissatisfied with the response of their employer/state to COVID-19, interacting with friends/family less than usual, and using social media more than usual, as well as those with a less than usual ability to share concerns with friends/family. Significantly higher scores on the SRQ (*P*<.05) were also seen in participants with pre-existing psychiatric conditions and previous exposure to traumatic experiences, and those who self-reported being a pessimist or introvert. Means and standard deviations for all comparisons are presented in Multimedia Appendix 10.

**Figure 4:**
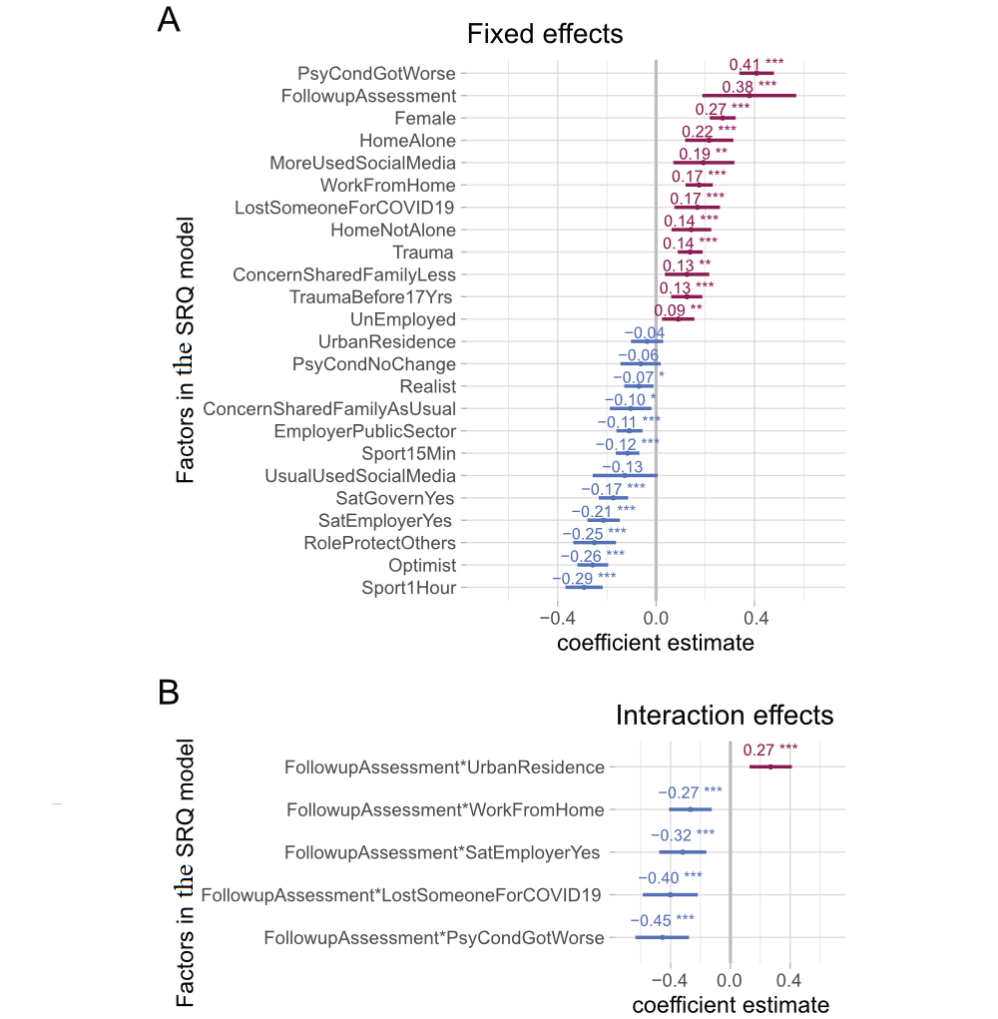
Factors associated with general psychological disturbance in the second assessment. These foster plots show the mean estimates and the 95% CIs for adjusted coefficients affecting the Self-Reporting Questionnaire-20 (SRQ) generated through multiple regression. Panel A shows fixed factors for SRQ scores during the second assessment. Panel B indicates interaction terms included in our regression model, indicating a significant difference between the fixed effects and SRQ scores during the first phase of the data collection and the second assessment. Factors increasing the SRQ score are shown in red, and factors decreasing the SRQ score are shown in blue.

Adjusted analysis utilizing a generalized linear model for the SRQ is reported in Figure 4. The following factors were independently associated with increased SRQ scores on the second assessment: a psychiatric condition that worsened during the COVID-19 pandemic (SRQ mean coefficient: 0.41, 95% CI 0.33-0.48), previous exposure to trauma before and after age 17 years (SRQ mean coefficient: 0.13, 95% CI 0.06-0.19 and SRQ mean coefficient: 0.14, 95% CI 0.08-0.19, respectively), and isolating at home alone (SRQ mean coefficient: 0.22, 95% CI 0.12-0.31). In addition, increased social media usage, working from home, and death of a family member due to COVID-19 significantly increased SRQ scores (SRQ mean coefficient: 0.19, 95% CI 0.07-0.32; SRQ mean coefficient: 0.17, 95% CI 0.12-0.23; and SRQ mean coefficient: 0.17, 95% CI 0.07-0.26). Moreover, significant gender differences were observed, with higher scores in women versus men (SRQ mean coefficient: 0.27, 95% CI 0.22-0.32). Having an optimistic attitude and feeling a sense of control in protecting oneself and others during the COVID-19 pandemic were associated with decreased SRQ scores in the second assessment, indicating a potentially protective effect of these factors against persistent general psychological disturbance (SRQ mean coefficient: −0.26, 95% CI −0.32 to −0.20 and SRQ mean coefficient: −0.25, 95% CI 0.12 to 0.23, respectively). Furthermore, participants who were satisfied with the employer/state response to COVID-19 and were able to share concerns with family/friends had lower SRQ scores overall (SRQ mean coefficient: −0.21, 95% CI −0.27 to −0.15; SRQ mean coefficient: 0.17, 95% CI −0.23 to −0.11; and SRQ mean coefficient: −0.10, 95% CI −0.19 to −0.02, respectively). Furthermore, daily physical activity/sports significantly decreased the SRQ score (mean coefficient: −0.29, 95% CI −0.37 to −0.22), with a greater protective effect associated with a higher duration of the physical activity/sport (exercise for ≥1 hour was more effective in decreasing the SRQ score compared to exercise for >15 minutes but <1 hour).

Finally, by including interaction terms in our regression model, we found that there was a relationship between residence type and SRQ score changes between the first and second assessments. Notably, SRQ scores increased in people living in urban areas compared to those living in rural areas (mean coefficient of interaction between acute/persistent and residence type: 0.27, 95% CI 0.13-0.41). Additionally, both people working from home and not working from home demonstrated a difference in responses between the two surveys (mean coefficient of interaction between acute/persistent and working from home: −0.27, 95% CI −0.41 to −0.12). Moreover, people who reported worsening of pre-existing psychiatric conditions during the first assessment reported lower SRQ scores in the second assessment, whereas those with no pre-existing psychiatric condition or a psychiatric condition that did not worsen showed an increase in SRQ scores in the second assessment (mean coefficient of interaction between acute/persistent and psychiatric condition: −0.45, 95% CI −0.64 to −0.28).

### Data and Material Availability

All data presented in the main text and supplementary items are deposited in a repository [36].

## Discussion

This study, performed on a global scale, highlights the significant impact of the COVID-19 pandemic on the mental health of internet users during the first wave of the pandemic when the strictest lockdown restrictions were in place. It also provides evidence for the presence of these effects in a population subset of European participants 1 month later when restrictions were comparably less strict.

A major aim of this study was to identify specific factors that were positively or negatively associated with psychological perturbations in the immediate aftermath of the COVID-19 pandemic. Notably, the study was conducted when the strictest lockdown measures were in place, and the internet became the default mode of personal and professional communication. Worsening of a pre-existing psychiatric condition, female gender identification, previous exposure to trauma, and working remotely were associated with higher risks for general psychological disturbance, PTSD, and depression. Additionally, considering oneself an introvert was associated with the heightened risk of general psychological disturbance and depression, as was being unemployed, living alone, and having limited interaction with family and friends. An overall protective effect against all major psychological conditions was observed for the following factors: increasing age, considering oneself an optimist, optimism about the COVID-19 pandemic outcome, ability to share concerns with family and friends like usual, daily physical exercise/sports for 15 minutes or more, and being satisfied with the actions of the employer and state in the response to COVID-19.

To ensure that the psychological symptoms assessed in this study were related to the COVID-19 pandemic, the participants were repeatedly prompted to consider COVID-19 and their feelings during the preceding week while filling in the survey. Furthermore, the phrase “this crisis” was present in all the screening questions, for example, “I am unable to sleep well during this crisis.” Further considerations about the attributability of psychological symptoms to the COVID-19 pandemic include the difference in the proportion (22%) of participants who reported pre-existing psychiatric conditions versus those who reported general psychological disturbance (43%) assessed through the SRQ during the first assessment. Furthermore, we compared the prevalence of depression in all of our featured countries based on different BDI cutoffs for depression versus the most recent available statistics from the WHO (2017) and found a remarkable difference. 

To the best of our knowledge, this study is one of the few worldwide assessments of the mental health effects of COVID-19 performed during the first global wave of COVID-19. Earlier studies on the psychological impact of the COVID-19 outbreak were mostly from China [9,17,35,37-42]. However, a large number of studies on pandemic-related psychopathology have since been published, mostly focusing on populations from specific cities or countries [43-45]. Nevertheless, assessments performed on a global scale have been accumulating [46-51]. A study of almost 30,000 individuals across four South Asian countries showed that anxiety and depression were more common in those with chronic diseases and lower socioeconomic status [47]. Another study (n=4612) across eight countries showed that excessive and contradictory health information related to COVID-19 contributed to the psychological effects reported during the pandemic [46]. Another study (n=7091) across 13 countries found that, in alignment with our results, female individuals were more likely to report distress during the pandemic [48]. Similarly, a study of 9565 participants from 78 different countries showed that social support, finances, and psychological flexibility were the strongest predictors of being psychologically impacted by the pandemic [50]. Finally, our results are in agreement with findings from a recent study performed across Europe (n=15,790), which showed that lack of social contact has been a major stressor for individuals during the pandemic [51].

Identification of specific factors that are associated with an increased or decreased susceptibility to being psychologically affected by the pandemic could be crucial to mitigate the negative mental health impact of the COVID-19 pandemic at regional and global levels. For example, the vulnerability of females indicated in this study warrants further investigation for both the contributing factors and the resulting implications of such an increased risk. These include social factors, such as increased reporting of domestic violence in relation to COVID-19 [52], possible caregiver stress, and the impact of changes in familial roles and responsibilities secondary to the current health emergency. Furthermore, an increased risk of psychological symptoms in individuals with pre-existing psychiatric conditions and/or previous trauma exposure necessitates the initiation and/or expansion of mental health support systems available remotely [53]. Emerging evidence now supports the efficacy of web and social media–based interventions in promoting mental health via paradigms based on mindfulness, positive psychology, and exercise [54-56]. Such interventions could be developed at the governmental and institutional levels and delivered to the general public via mainstream and social media. Indeed, media outlets could play a major role in promoting optimism and a positive attitude toward COVID-19 resolution, both of which were identified in our study as important resilience factors. Furthermore, the association between remote working and increased psychological symptoms calls for optimization of the work-from-home model and a greater emphasis on the general well-being of employees. This is further corroborated by the observation in this study that participant satisfaction with the employer response to the COVID-19 pandemic is associated with reduced psychological symptoms.

Specifically, the results of this global online survey could be beneficial in the optimization of digital mental health services tailored to the needs of the target populations. Importantly, the study included lower- and middle-income countries such as Pakistan, Iran, and Bosnia and Herzegovina, where telemedicine/telepsychiatry services are likely less represented. The results of our study could help facilitate the organization and implementation of such services and their delivery to vulnerable populations. Furthermore, the administered measures in our study allowed for simultaneous screening of some psychiatric comorbidities that were found to be correlative to one another. These findings can provide invaluable insights for improving digital mental health services, whereby the presence of one psychopathology could prompt screening for the other. Regarding the optimization of digital mental health services, it is also important to note that the availability of the questionnaire in 11 different languages in this study provides insights to help extrapolate the results to individuals using web tools in different languages. Furthermore, the timing of this study is an important strength. The initial assessment was performed from March 29 to April 14, 2020. This timing coincides with the peak of the COVID-19 pandemic in North America and Europe, a time when almost one-half of the world remained in complete lockdown [57]. The second assessment, targeted at participants in Europe, was performed after a 1-month period when the situation had improved considerably in Europe and lockdown measures had been relatively eased. Finally, the identification of resilience factors identified in this study could have implications for digital mental health services. For example, the protective effect of exercise calls for efforts to promote exercise and physical activity through web-based outlets such as mobile health apps. Similarly, the protective effects of having a positive prediction and an optimistic attitude about the resolution of the COVID-19 pandemic should be taken into consideration when designing applications for information related to the pandemic or general promotion of positive psychology. The protective effect of maintaining contact with friends and family could also be incorporated into such resources. As an example, health apps that allow individuals to coordinate physical exercise with their friends and/or family members could be designed. It is noteworthy that a volunteer-based telehealth program for supporting the mental health of the elderly during the COVID-19 pandemic referred to many of our findings, including those related to pre-existing psychiatric conditions and impact on the elderly [58].

The study has potential limitations that warrant consideration when interpreting the results. First, the study employed a nonrandomized sampling strategy, and we advise caution in generalizing the findings of the study. The disproportionate demographic representation combined with the online nature of the study raises the potential for some level of participation bias. The association between self-reports of increased social media use and increased psychological symptoms must be interpreted with caution considering the study itself was conducted online. Furthermore, the unexpected result regarding medical or health care professionals reporting lower SRQ scores than the rest of the participants could have resulted from the disproportionate demographic representation; only 102 medical professionals participated in our survey.

The second considerable limitation is the use of self-report scales rather than clinical verification. However, the anonymous nature of the survey and widespread social distancing measures precluded such verification. Additionally, it is not possible to adjust for the confounding effect of non-COVID-19-related individual crisis situations on participant responses. We tried to reduce this effect by formatting survey questions in such a way that would prompt participants to consider their mental state specific to the COVID pandemic. Finally, any interpretation of the results from the second assessment warrants even more caution, as (1) the anonymous nature of the survey prevents verification of whether these are the same participants who filled the first survey; (2) it is unclear if the symptoms have persisted or are newly developed in the interval between the two assessments; and (3) only a fraction of individuals took part in the second assessment despite repeated reminders, leaving a viable possibility of participation bias.

In conclusion, this effort highlights the impact of the COVID-19 pandemic at both the regional and global levels on the mental health of internet users. Further, our study elucidates prominent associations with participant demographics, history of psychiatric disease risk factors, household conditions, personality traits, and attitudes toward COVID-19. These results could serve to inform health professionals and policymakers across the globe, aiding in dynamic optimization of digital mental health services during and following the COVID-19 pandemic, and potentially reducing its long-term morbidity and mortality.

## Appendix

Multimedia Appendix 1 Study questionnaire.

Multimedia Appendix 2 Switzerland ethical approval.

Multimedia Appendix 3 Poland ethical approval.

Multimedia Appendix 4 Bosnia and Herzegovina ethical approval.

Multimedia Appendix 5 Number of participants per country and World Health Organization region included in the primary assessment.

Multimedia Appendix 6 Demographics and characteristics of the participants included in the primary assessment.

Multimedia Appendix 7 Predictors for acute general psychological disturbance, posttraumatic stress disorder risk, and depression.

Multimedia Appendix 8 Association of participant demographics/characteristics and categorical classifications for general psychological disturbance (Self-Reporting Questionnaire-20), posttraumatic stress disorder risk (Impact of Event Scale), and depression (Beck Depression Inventory II) in the primary assessment.

Multimedia Appendix 9 Correlations between the Self-Reporting Questionnaire-20, Impact of Event Scale, and Beck Depression Inventory II scores for the primary assessment.

Multimedia Appendix 10 Comparison of psychological symptoms between different participant demographics/characteristics included in the follow-up assessment.

## Abbreviations

BDI: Beck Depression Inventory II
CET: Central European time
IES: Impact of Event Scale
OR: odds ratio
PTSD: posttraumatic stress disorder
SRQ: Self-Reporting Questionnaire-20
WHO: World Health Organization
