# Supplementary material for: Factors Associated With Psychological Disturbances During the COVID-19 Pandemic: Multicountry Online Study
Source: JMIR Ment Health. 2021 Aug 19;8(8):e28736. doi: 10.2196/28736 (PMC8396308; doi:10.2196/28736)
Supplement: Multimedia Appendix 2 [file mental_v8i8e28736_app2.pdf]

Kanton Zürich
Kantonale Ethikkommission

Prof. Dr. med. Peter Meier-Abt
Präsident

Dr. med. Peter Kleist
Geschäftsführer
Stampfenbachstrasse 121
Postfach
8090 Zürich
Telefon +41 43 259 79 70
Fax +41 43 259 79 72
www.kek.zh.ch

Brain Research Institute
Dr. Ali Jawaid
Dörflistrasse. 67. 401
8050 Zurich

16. April 2020 / ktr
BASEC-Nr. Req-2020-00419
Clarification of responsibility

Title: It is an anonymous global survey on mental health impact of COVID-19. The results of this survey could be invaluable in informing health systems about the impact of COVID-19 and its associated factors, such as social disconnectedness on mental health of masses.

Dear Doctor Jawaid

We refer to your submission dated 11.04.2020.

Your research project does not fall within the scope of the Human Research Act (HRA). Therefore, an authorization from the ethics committee is not required.

Kindly note that an invoice in the amount of CHF 200.- will be issued by the cantonal accounts department.

Sincerely yours,

Peter Kleist
