# Supplementary material for: Factors Associated With Psychological Disturbances During the COVID-19 Pandemic: Multicountry Online Study
Source: JMIR Ment Health. 2021 Aug 19;8(8):e28736. doi: 10.2196/28736 (PMC8396308; doi:10.2196/28736)
Supplement: Multimedia Appendix 3 [file mental_v8i8e28736_app3.pdf]

NENCKI INSTITUTE OF EXPERIMENTAL BIOLOGY
Laboratory for Neurobiology
02-093 Warsaw, Pasteura 3, Poland

phone: (48-22) 659 30 01
fax: (48-22) 822 53 42
http://neurogene.nencki.gov.pl

Leszek Kaczmarek Ph.D.
Professor and Head, BRAINCITY President
Member: Polish Academy of Sciences, EMBO, Academia Europaea
Email: l.kaczmarek@nencki.edu.pl

April 3rd 25, 2020

To whom it may concern,

With this letter, I am expressing mu full support for the project “Mental health impact of COVID-19: A risk and resilience study” led by Dr. Ali Jawaid, the newly recruited Senior Group Leader at the BRAINCITY: Center of Excellence for Neural Plasticity & Brain Disorders, recently created at the Nencki Institute as the European Molecular Biology (EMBL) partnership.

This global study aiming at screening for psychological and psychosomatic manifestations of depression, anxiety, and post-traumatic stress disorder (PTSD) among people that are either directly or indirectly affected by the COVID-19 pandemicis is very timely, important and shall bring significant results regarding possible long-lasting debilitating consequences of the present situation.

The results of the proposed, fully anonymous, survey could be invaluable in informing health systems about the impact of COVID-19 pandemic and its associated factors, such as social disconnectedness, stress, disrupted routine etc. on mental health of large populations of people.

Leszek Kaczmarek, BRAINCITY President
