# Supplementary material for: Factors Associated With Psychological Disturbances During the COVID-19 Pandemic: Multicountry Online Study
Source: JMIR Ment Health. 2021 Aug 19;8(8):e28736. doi: 10.2196/28736 (PMC8396308; doi:10.2196/28736)
Supplement: Multimedia Appendix 4 [file mental_v8i8e28736_app4.pdf]

UNIVERSITY OF TUZLA

Faculty of Medicine

Univerzitetska 1, 75000 Tuzla

Bosnia and Herzegovina

To whom it may concern,

With this letter, I am expressing my full support for the project “Mental health impact of COVID- 19: A risk and resilience study” led by Dr. Ali Jawaid, currently a group leader at University of Zurich, Switzerland and the incoming senior group leader at the BRAINCITY: Center of Excellence for Neural Plasticity & Brain Disorders, Nencki Institute of Experimental Biology, Warsa, Poland.

This global study aiming at screening for psychological and psychosomatic manifestations of depression, anxiety, and post-traumatic stress disorder (PTSD) among people that are either directly or indirectly affected by the COVID-19 pandemicis is very timely, important and shall bring significant results regarding possible long-lasting debilitating consequences of the present situation.

The results of the proposed, fully anonymous, survey could be invaluable in informing health systems about the impact of COVID-19 pandemic and its associated factors, such as social disconnectedness, stress, disrupted routine etc. on mental health of large populations of people and could have important implications for psychiatry/psychology prevention and therapeutics. We are glad that a number of our students are involved in this study under supervision of Dr. Jawaid.

Dean of Faculty of Medicine

Prof.dr.sc Selmira Brkić
