# Supplementary material for: Factors Associated With Psychological Disturbances During the COVID-19 Pandemic: Multicountry Online Study
Source: JMIR Ment Health. 2021 Aug 19;8(8):e28736. doi: 10.2196/28736 (PMC8396308; doi:10.2196/28736)
Supplement: Multimedia Appendix 5 [file mental_v8i8e28736_app5.docx]

**Multimedia Appendix 5.** Number of participants per country and World Health Organization region included in the primary assessment.

|  | N |
| --- | --- |
| PAHO | 254 |
| SEARO | 259 |
| EURO | 784 |
| EMRO | 459 |
| WPRO | 326 |
| Bosnia and Herzegovina | 885 |
| Canada | 538 |
| France | 337 |
| Germany | 534 |
| Iran | 1198 |
| Italy | 1096 |
| Pakistan | 1773 |
| Poland | 1110 |
| Spain | 972 |
| Switzerland | 489 |
| Turkey | 539 |
| United States | 1864 |
