# Supplementary material for: Factors Associated With Psychological Disturbances During the COVID-19 Pandemic: Multicountry Online Study
Source: JMIR Ment Health. 2021 Aug 19;8(8):e28736. doi: 10.2196/28736 (PMC8396308; doi:10.2196/28736)
Supplement: Multimedia Appendix 6 [file mental_v8i8e28736_app6.docx]

**Multimedia Appendix 6.** Demographics and characteristics of the participants included in the primary assessment.

| Predictor |  | n | % |
| --- | --- | --- | --- |
| Gender | Male | 3235 | 25.13 |
|  | Female | 9314 | 72.36 |
|  | Non-binary | 92 | 0.71 |
|  | Not disclosed | 92 | 0.71 |
| Residence | Rural | 1981 | 15.39 |
|  | Urban | 10666 | 82.87 |
| Education | Compulsory | 2980 | 23.15 |
|  | Advanced | 9653 | 75.00 |
| Work Status | Private employed | 2483 | 19.29 |
|  | Public employed | 2326 | 18.07 |
|  | Freelancer | 1003 | 7.79 |
|  | Unemployed | 3020 | 23.46 |
| Medical or healthcare professional | No | 11680 | 90.75 |
|  | Yes | 1020 | 7.92 |
| Remotely working from home | No | 4437 | 34.47 |
|  | Yes | 8289 | 64.40 |
| Opinion about employer response to COVID-19 | Not satisfied with employer | 1412 | 10.97 |
|  | Somewhat satisfied with employer | 2369 | 18.41 |
|  | Satisfied with employer | 4364 | 33.91 |
| Opinion about state response to COVID-19 | Not satisfied with employer | 3948 | 30.67 |
|  | Somewhat satisfied with government | 4772 | 37.08 |
|  | Satisfied with government | 4009 | 31.15 |
| Home Isolation | No home isolation | 1002 | 7.78 |
|  | Home isolation (alone at home) | 1063 | 8.26 |
|  | Home with family or partner | 10691 | 83.06 |
| Presence of pet at home | No pet at home | 8318 | 64.63 |
|  | Pet at home | 4390 | 34.11 |
| Interaction with family or friends | Less than usual | 4082 | 31.71 |
|  | Minimal interaction | 3641 | 28.29 |
|  | Like usual | 4707 | 36.57 |
| Use of social media | Less than usual | 685 | 5.32 |
|  | Like usual | 3635 | 28.24 |
|  | More than usual | 8385 | 65.15 |
| Time dedicated to physical exercise | Less than 15 minutes | 6306 | 48.99 |
|  | More than 15 minutes | 4670 | 36.28 |
|  | More than 1 hour | 1709 | 13.28 |
| Close person positive for COVID-19 | No | 9434 | 73.30 |
|  | Yes | 3281 | 25.49 |
| Close person demised due to COVID-19 | No | 11761 | 91.38 |
|  | Yes | 976 | 7.58 |
| Psychiatric Condition | No psychiatric condition | 10010 | 77.77 |
|  | Pre-existing psychiatric condition no change | 1053 | 8.18 |
|  | Pre-existing psychiatric condition got worse | 1693 | 13.15 |
| Ability to share concerns with health professional | Yes | 4934 | 38.33 |
|  | No | 2929 | 22.76 |
| Ability to share concerns with family or friends | Not at all | 1098 | 8.53 |
|  | Less than usual | 2674 | 20.78 |
|  | Like usual | 8924 | 69.33 |
| Previous exposure to crisis | No | 9581 | 74.44 |
|  | Yes | 3116 | 24.21 |
| Previous exposure to traumatic experiences | No | 6740 | 52.37 |
|  | Yes | 4330 | 33.64 |
|  | Yes (before the age of 17) | 1686 | 13.10 |
| Personality | Extrovert | 5369 | 41.71 |
|  | Introvert | 6652 | 51.68 |
| Personality | Pessimist | 2060 | 16.00 |
|  | Optimist | 5181 | 40.25 |
|  | Realist | 5450 | 42.34 |
| Prediction about COVID-19 outcome/  resolution | It might be the end of human race | 138 | 1.07 |
|  | It will resolve after many months or years | 3819 | 29.67 |
|  | It will resolve in the summer but not within a month | 7114 | 55.27 |
|  | It will resolve within a month | 1038 | 8.06 |
| Self-opinion in COVID-19 pandemic | It is not in my control at all | 471 | 3.66 |
|  | It is not in my control but I can take precautions to protect myself | 1782 | 13.85 |
|  | It is not in my control but I can take precautions to protect myself and also others | 10408 | 80.86 |
