# Supplementary material for: Factors Associated With Psychological Disturbances During the COVID-19 Pandemic: Multicountry Online Study
Source: JMIR Ment Health. 2021 Aug 19;8(8):e28736. doi: 10.2196/28736 (PMC8396308; doi:10.2196/28736)
Supplement: Multimedia Appendix 7 [file mental_v8i8e28736_app7.docx]

**Multimedia Appendix 7.** Predictors for acute general psychological disturbance, posttraumatic stress disorder risk, and depression.

|  | SRQ |
| --- | --- |
| Pre-existing Psychiatric Condition Got Worse | 5.28 *** [4.30,6.49] |
| Previous Exposure to Trauma | 1.95 *** [1.62,2.34] |
| More Usage Social Media | 1.35 [0.90,2.02] |
| Previous Exposure to Trauma Before 17Yrs | 1.25 [0.98,1.59] |
| Concern Shared with Family Less | 1.03 [0.76,1.39] |
| Pre-existing Psychiatric Condition | 0.64 ** [0.49,0.84] |
| Physical exercise/Sport 15Min | 0.65 *** [0.54,0.77] |
| Used Usage of Social Media | 0.64 * [0.41,0.98] |
| Realist | 0.56 *** [0.44,0.70] |
| Physical exercise/Sport 1Hour | 0.51 *** [0.39,0.67] |
| Optimist | 0.38 *** [0.30,0.48] |
| Concern Shared with Family as Usual | 0.38 *** [0.29,0.50] |
| N | 3635 |
| BIC | 3790.10 |
| *** p < 0.001; ** p < 0.01; * p < 0.05. | |

|  | IES |
| --- | --- |
| Pre-existing Psychiatric Condition Got Worse | 1.60 *** [1.38,1.84] |
| Previous Exposure to Crisis | 1.16 ** [1.05,1.29] |
| Introvert | 0.96 [0.88,1.04] |
| Pet | 0.94 [0.85,1.03] |
| Pre-existing Psychiatric Condition | 0.87 [0.74,1.01] |
| N | 11953 |
| BIC | 12546.98 |
|  |  |
| *** p < 0.001; ** p < 0.01; * p < 0.05. | |

|  | BDI |
| --- | --- |
| Pre-existing Psychiatric Condition Got Worse | 7.10 *** [6.03,8.35] |
| Previous Exposure to Trauma | 1.61 *** [1.46,1.76] |
| Previous Exposure to Trauma Before 17Yrs | 1.28 *** [1.13,1.45] |
| Concern Shared with Family Less | 1.23 * [1.04,1.46] |
| More Used Social Media | 1.04 [0.87,1.26] |
| Pre-existing Psychiatric Condition | 1.02 [0.89,1.18] |
| Usual Usage Social Media | 0.72 *** [0.59,0.88] |
| Realist | 0.41 *** [0.37,0.47] |
| Concern Shared with Family As Usual | 0.39 *** [0.33,0.45] |
| Optimist | 0.23 *** [0.20,0.26] |
| N | 12554 |
| BIC | 14118.74 |
| *** p < 0.001; ** p < 0.01; * p < 0.05. | |

Logistic regression was performed to generate odds ratios (ORs) for SRQ, IES, and BDI using the following categorization scheme; SRQ: 0 = normal (0-7 points), 1 = concern for general psychological disturbance (8-20 points); IES: 0 = normal (0-23 points), 1 = PTSD is a clinical concern (24-32 points), 2 = threshold for a probable PTSD diagnosis (33-36 points), 3 = Severe condition (high enough to induce immunosuppression) (37+ points). For generating ORs, the variables were regrouped as 0 = no concern versus any type of concern (1/2/3); BDI: 0 = These ups and downs are considered normal (1-10 points). 1 = Mild mood disturbance (11-16 points), 2 = Borderline clinical depression. (17-20 points), 3 = Moderate Depression (21-30 points), 4 = Severe Depression (31-40 points), 5 = Extreme Depression (>40 points). For generating ORs, the variables were regrouped as 0 = no concern versus any type of concern (levels 1/2/3/4/5). Only the factors that survived step BIC models comparison are listed. OR >1 indicate increased risk and OR<1 indicates protective effect.
