# Supplementary material for: Factors Associated With Psychological Disturbances During the COVID-19 Pandemic: Multicountry Online Study
Source: JMIR Ment Health. 2021 Aug 19;8(8):e28736. doi: 10.2196/28736 (PMC8396308; doi:10.2196/28736)
Supplement: Multimedia Appendix 8 [file mental_v8i8e28736_app8.docx]

**Multimedia Appendix 8.** Association of participant demographics/characteristics and categorical classifications for general psychological disturbance (Self-Reporting Questionnaire-20), posttraumatic stress disorder risk (Impact of Event Scale) and depression (Beck Depression Inventory II) in the primary assessment.


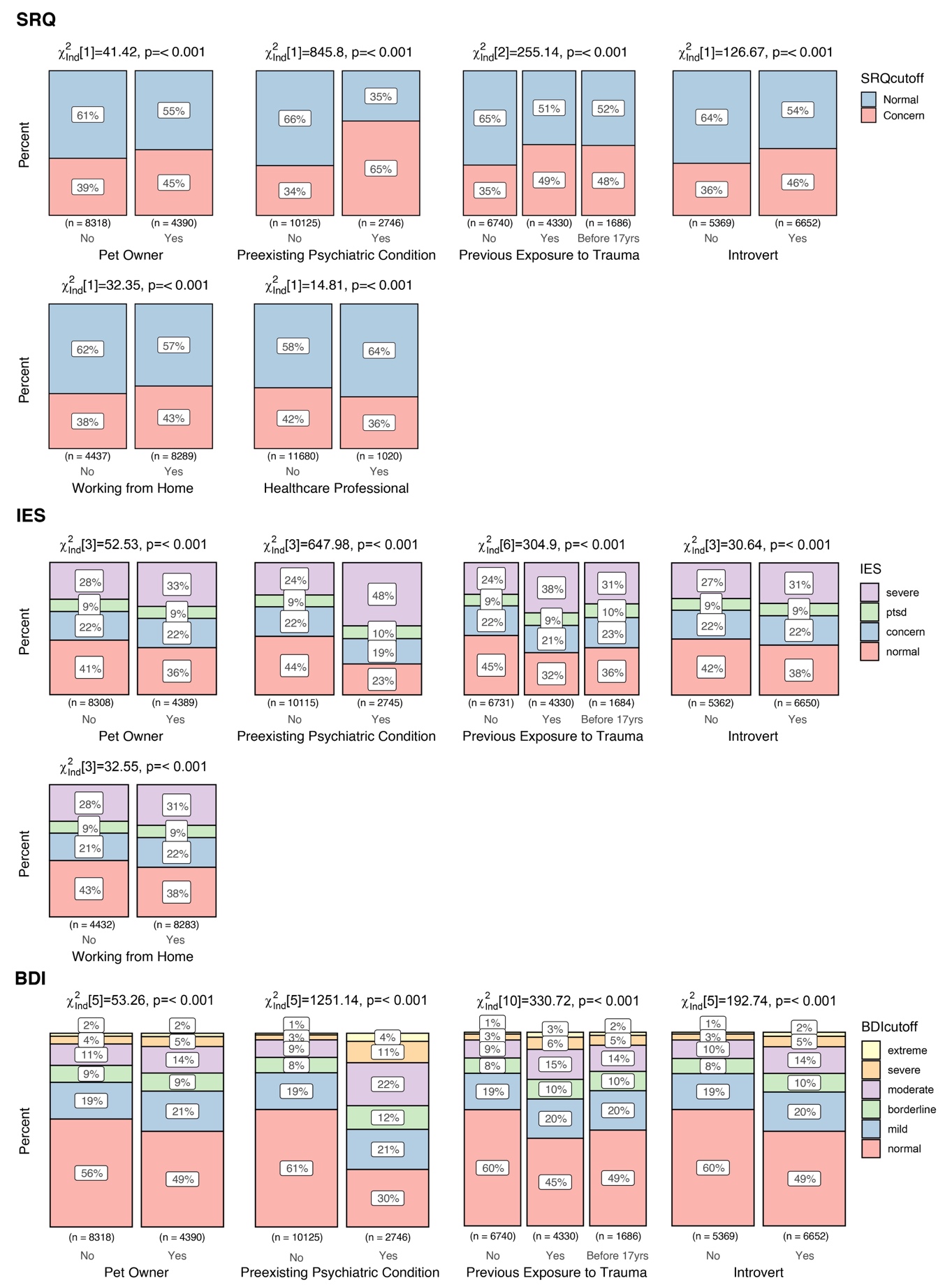


These tables show results from the predictors with a significant chi-square value after correcting for multiple comparisons (Bonferroni correction for 27 tests, p<0.0019). For each predictor, the bar plot indicates the participant’s distribution across the different categories of SRQ, IES, and BDI. The cut-offs used are as follows: for SRQ normal/concern (0-7, 8-20 points); for IES normal/concern/PTSD/severe (0-23, 24-32, 33-36, 37+ points); for BDI normal/mild/borderline/moderate/severe/extreme (1-10, 11-16, 17-20, 21-30, 31-40, 40+ points). The plots include the total number and %age of participants in each category and the statistical outcomes from the chi-square test.
