# Supplementary material for: Factors Associated With Psychological Disturbances During the COVID-19 Pandemic: Multicountry Online Study
Source: JMIR Ment Health. 2021 Aug 19;8(8):e28736. doi: 10.2196/28736 (PMC8396308; doi:10.2196/28736)
Supplement: Multimedia Appendix 9 [file mental_v8i8e28736_app9.docx]

**Multimedia Appendix 9.** Correlations between the Self-Reporting Questionnaire-20, Impact of Event Scale, and Beck Depression Inventory II scores for the primary assessment.


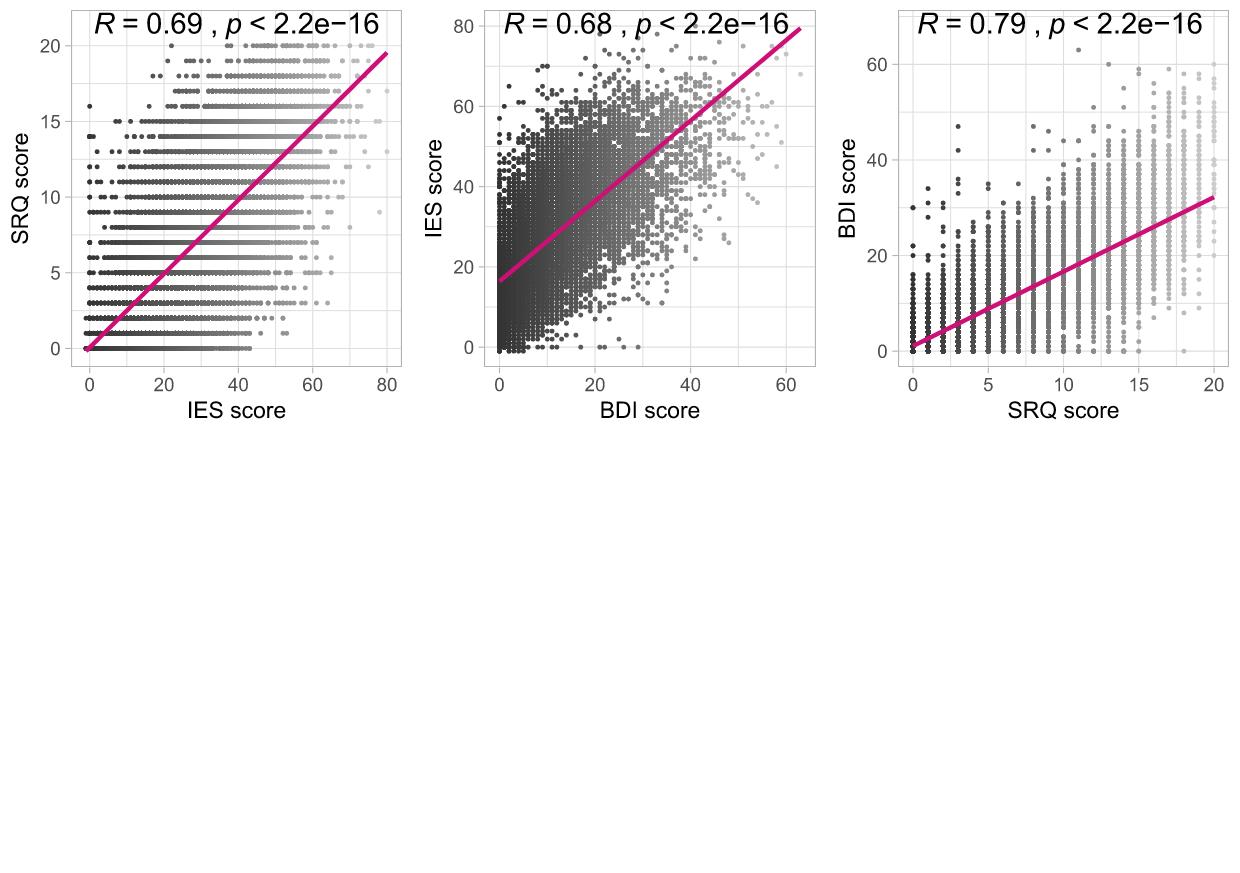


These plots show Pearson’s correlation values for all the possible pairs of the three scales used, i.e., SRQ vs. IES, IES vs. BDI, and BDI vs. SRQ. All the correlations are statistically significant and range from 68% to 79% correlation.
