# Supplementary material for: Factors Associated With Psychological Disturbances During the COVID-19 Pandemic: Multicountry Online Study
Source: JMIR Ment Health. 2021 Aug 19;8(8):e28736. doi: 10.2196/28736 (PMC8396308; doi:10.2196/28736)
Supplement: Multimedia Appendix 10 [file mental_v8i8e28736_app10.docx]

**Multimedia Appendix 10.** Comparison of psychological symptoms between different participant demographics/characteristics included in the follow-up assessment.

| Predictors |  | SRQ |
| --- | --- | --- |
| Gender | Male | **6.03 ± 4.82** |
|  | Female | **8.24 ± 4.86** |
|  | Non-binary | **6.60 ± 5.25** |
|  | Not disclosed | **4.50 ± 4.82** |
| Residence | Rural | 6.98 ± 5.02 |
|  | Urban | 7.08 ± 5.06 |
| Education | Primary | 7.27 ± 2.59 |
|  | Secondary | 7.10 ± 5.07 |
|  | Bachelor | 7.23 ± 4.95 |
|  | Master | 7.45 ± 4.84 |
|  | PhD | 6.96 ± 4.44 |
| Working position | Private employed | 6.22 ± 5.00 |
|  | Public employed | 6.15 ± 4.89 |
|  | Freelancer | 6.79 ± 4.45 |
|  | Unemployed | 8.17 ± 5.03 |
| Medical or healthcare professional | No | **7.32** ± **4.87** |
|  | Yes | **9.15** ± **4.82** |
| Work from home | No | 7.60 ± 4.72 |
|  | Yes | 7.75 ± 4.97 |
| Opinion on employer attitude | Not satisfied | **9.84 ± 4.83** |
|  | Somewhat satisfied | **7.79 ± 4.89** |
|  | Satisfied | **5.78 ± 4.94** |
| Opinion on governament attitude | Not satisfied | **8.48 ± 4.84** |
|  | Somewhat satisfied | **7.66 ± 4.76** |
|  | Satisfied | **6.37 ± 5.07** |
| Home Isolation | Not isolated | 6.85 ± 4.77 |
|  | Individual home isolation | 8.08 ± 4.87 |
|  | Home isolation with family or partner | 7.19 ± 5.49 |
| Presence of pet at home | No pet at home | 7.39 ± 4.81 |
|  | Pet at home | 8.21 ± 4.96 |
| Possibility to interact with family or friends | Less than usual | **7.83 ± 5.02** |
|  | Minimal interaction | **9.44 ± 4.81** |
|  | Like usual | **6.42 ± 4.75** |
| Use of social media | Less than usual | **8.45 ± 4.74** |
|  | Like usual | **4.98 ± 5.24** |
|  | More than usual | **8.97 ± 4.47** |
| Time dedicated to physical exercise | Less than 15 minutes | **8.38 ± 5.13** |
|  | More than 15 minutes | **5.95 ± 4.85** |
|  | More than 1 hour | **5.94 ± 4.71** |
| Close person positive for COVID-19 | No | **6.62 ± 4.91** |
|  | Yes | **8.99 ± 4.82** |
| Close person dead for COVID-19 | No | **7.28 ± 4.89** |
|  | Yes | **9.44 ± 4.73** |
| Psychiatric Condition | Absence of psychiatric disorders | **7.25 ± 4.84** |
|  | No changes in pre-existing psychiatric disorders | **6.86 ± 4.69** |
|  | Worsening of pre-existing psychiatric disorders | **11.84 ± 5.12** |
| Possibility to share concerns with professional | No | 8.67 ± 4.91 |
|  | Yes | 8.70 ± 5.04 |
| Possibility to share concerns with family or friends | No | **9.89 ± 4.80** |
|  | Less than usual | **6.81 ± 4.90** |
|  | Like usual | **6.43 ± 4.61** |
| Previous exposure to crisis | No | 7.68 ± 4.77 |
|  | Yes | 7.76 ± 5.21 |
| Previous exposure to traumatic experiences | No | **6.88 ± 4.75** |
|  | Yes | **9.44 ± 5.02** |
|  | Yes (before 17 years old) | **7.70 ± 4.87** |
| Personality | Extrovert | **7.14 ± 4.69** |
|  | Introvert | **8.47 ± 5.06** |
| Personality | Pessimist | **9.54 ± 5.01** |
|  | Optimist | **6.07 ± 4.88** |
|  | Realist | **7.38 ± 4.91** |
| Prediction about the resolution of COVID-19 pandemic | It might be the end of human race | 7.95 ± 3.96 |
|  | It will resolve after many months or years | 8.07 ± 4.88 |
|  | It will resolve in the summer but not within a month | 6.98 ± 4.82 |
|  | It will resolve within a month | 7.43 ± 5.18 |
| Self-opinion in COVID-19 pandemic | It is not in my control at all | 7.62 ± 4.36 |
|  | It is not in my control but I can take precautions to protect myself | 8.00 ± 4.87 |
|  | It is not in my control but I can take precautions to protect myself and also others | 7.78 ± 4.88 |

This table shows means ± standard deviations of participants’ SRQ scores divided according to different participant demographics/ characteristics and compared through unadjusted Kruskal- Wallis tests. Significant differences (p-value threshold set to <0.05) in mean scores are highlighted as bold. Each bold association indicates difference in categories reported in the predictors’ column vertically.
